# Activation of NLR-Mediated Autoimmunity in Arabidopsis *Early in Short Days 4* Mutant

**DOI:** 10.3389/fpls.2022.881212

**Published:** 2022-05-25

**Authors:** Xingchuan Huang, Yanan Liu, Jianhua Huang, W. G. Dilantha Fernando, Xin Li, Shitou Xia

**Affiliations:** ^1^Hunan Provincial Key Laboratory of Phytohormones and Growth Development, College of Bioscience and Biotechnology, Hunan Agricultural University, Changsha, China; ^2^Michael Smith Laboratories, University of British Columbia, Vancouver, BC, Canada; ^3^Department of Botany, University of British Columbia, Vancouver, BC, Canada; ^4^Department of Plant Science, University of Manitoba, Winnipeg, MB, Canada

**Keywords:** *ESD4*, SUMO modification, NHP signaling, *EDS1*, NLR, autoimmunity

## Abstract

From a reverse genetic screen using CRISPR/Cas9 gene editing tool, we unintentionally identified an autoimmune mutant. Map-based cloning and whole-genome sequencing revealed that it contains a deletion in SMALL UBIQUITIN-RELATED MODIFIER (SUMO) protease encoding gene *EARLY IN SHORT DAYS 4* (*ESD4*). Previous studies reported that *esd4* mutants accumulate elevated levels of plant defense hormone salicylic acid (SA). However, upregulated *PATHOGENESIS-RELATED GENE 1* (*PR1*) expression in *esd4* only partly relies on SA level. In this study, we show that plant metabolite N-hydroxypipecolic acid (NHP) biosynthetic genes are upregulated in *esd4*, and NHP biosynthesis mutant *flavin-dependent-monooxygenase 1* (*fmo1*) partially suppresses the autoimmune phenotypes of *esd4*, suggestive of a requirement of NHP signaling for the autoimmunity in *esd4*. As activation of nucleotide-binding leucine-rich repeat immune receptors (NLRs) are associates with the biosynthesis of SA and NHP and lipase-like protein ENHANCED DISEASE SUSCEPTIBILITY 1 (EDS1) is a key component downstream of many NLRs, we examined the relationship between EDS1 and ESD4 by analyzing the *eds1 esd4* double mutant. We found that *eds1* largely suppresses *esd4* autoimmunity and blocks the elevated expressions of SA and NHP biosynthesis-related genes in *esd4*. Overall, our study provides evidence supporting the hypothesis that SUMO protease ESD4 likely targets a yet to be identified guardee of NLR by removing its SUMO modification to avoid recognition by the cognate NLR. Loss of ESD4 results in activation of NLR-mediated autoimmunity.

## Introduction

To defend themselves against pathogen infections, plants have evolved a complicated immune system which mainly relies on two types of immune receptors: plasma membrane-localized pattern recognition receptors (PRRs), and intracellular nucleotide-binding leucine-rich repeat immune receptors (NLRs; Dangl and Jones, 2001; Jones and Dangl, 2006). PRRs are receptor-like kinases (RLKs) or receptor-like proteins (RLPs), which recognize conserved microbial features, pathogen-associated molecular patterns (PAMPs), leading to pattern-triggered immunity (PTI). However, pathogens can deliver effectors into intracellular space to inhibit PTI, thereby facilitating pathogenesis. To counteract, intracellular NLRs can directly or indirectly recognize effectors to trigger a stronger defense response termed effector-triggered immunity (ETI), which is often associated with programmed cell death to activate the hypersensitive response (HR; Nishimura and Dangl, 2010; Cui et al., 2015).

Plant NLRs are divided into three classes based on their N-terminal domains: CNLs (CC-NLRs) have N-terminal coiled-coil domains, TNLs (TIR-NLRs) contain N-terminal Toll/interleukin-1 receptor domain (TIR) and RNLs (RPW8-NLRs) have N-terminal coiled-coil domains similar to RESISTANCE TO POWDERY MILDEW 8 (Maruta et al., 2022). TNLs and CNLs function as “sensor” NLRs to sense pathogenic effectors or their modifications on host targets, while RNLs are required for immune signaling and cell death induction mediated by TNLs and CNLs, and thus are considered as “helper” NLRs. ENHANCED DISEASE SUSCEPTIBILITY 1 (EDS1), a lipase-like protein, is an important hub downstream of TNLs and a few CNLs (Falk et al., 1999; Lapin et al., 2020). In parallel, a glycosylphosphatidylinositol-anchored plasma membrane-localized protein, NON-RACE-SPECIFIC DISEASE RESISTANCE (NDR1), was shown to be required for the signaling of CNLs (Century et al., 1997; Aarts et al., 1998; Shapiro and Zhang, 2001; Knepper et al., 2011). How EDS1 and NDR1 regulate NLR-mediated signaling is still unclear.

Activation of NLRs induces a series of downstream defense responses including the generations of the phenolic defense hormone salicylic acid (SA) and N-hydroxypipecolic acid (NHP), which are required for both PTI/ETI at local sites and induced immunity in distal tissues. In *Arabidopsis thaliana*, pathogen-induced SA is mainly synthesized from chorismate by ISOCHORISMATE SYNTHASE 1 (ICS1) and AVRPPHB SUSCEPTIBLE 3 (PBS3; Rekhter et al., 2019; Torrens-Spence et al., 2019). ENHANCED DISEASE SUSCEPTIBILITY 5 (EDS5) is responsible for exporting SA precursor from plastids to the cytosol (Rekhter et al., 2019). Whereas, NHP is biosynthesized from L-lysine *via* a series of biochemical reactions catalyzed by enzymes including ABERRANT GROWTH AND DEATH2 (AGD2)-LIKE DEFENSE RESPONSE PROTEIN 1(ALD1; Song et al., 2004a,b; Navarova et al., 2012), SAR DEFICIENT 4 (SARD4; Ding et al., 2016; Hartmann et al., 2017) and FLAVIN-DEPENDENT MONOOXYGENASE 1 (FMO1; Chen et al., 2018; Hartmann et al., 2018).

SUMOylation, one of the post-translational protein modifications (PTMs), is conserved in all eukaryotes (Miura et al., 2007). SUMOylation is the process of attaching SUMO (small ubiquitin-like modifier) to target lysine residues of protein *via* a series of biochemical reactions facilitated by SUMO-activating enzyme (E1), the SUMO-conjugating enzyme (E2), and the SUMO E3 ligase. Firstly, SUMO is activated by E1 in an ATP-dependent manner to form a thioester bond between the carboxyl group of glycine in SUMO and the sulfhydryl group of the catalytic cysteine residue in E1. Activated SUMO is subsequently transferred to a cysteine residue in E2. Finally, E3 ligase aids the transfer of SUMO from E2 to the target substrate. It is worth mentioning that SUMOylation of a target protein can occur without the help of E3 ligases (Wilkinson and Henley, 2010). SUMOylation is a reversible and highly dynamic modification due to the presence of SUMO proteases (Yates et al., 2016). On one hand, SUMO proteases possess activity to release free SUMO from the target protein. On the other hand, they are responsible for the generation of mature SUMO by cleaving off the C-terminus of immature SUMO.

The diverse roles of SUMOylation in plant immune signaling have been emerging during the past few years (He et al., 2017; Verma et al., 2018). For instance, flagellin perception induces the SUMOylation of its receptor FLAGELLIN-SENSITIVE 2 (FLS2) through the degradation of SUMO protease Desi3a, resulting in the release of BOTRYTIS-INDUCED KINASE1 (BIK1) and the activation of downstream immune signaling (Orosa et al., 2018). Recently, it was shown that the SUMOylation of transcriptional factor WRKY33 controlled by two SUMO proteases SPF1/SPF2 facilitates the activation of downstream immune-related genes (Verma et al., 2021).

Arabidopsis SUMO protease ESD4 was identified due to its early flowering phenotype especially under short photoperiods (Reeves et al., 2002; Pineiro et al., 2003). In this study, we show that loss of ESD4 results in autoimmunity including upregulated defense-related genes, spontaneous cell death, highly accumulated H_2_O_2_, and enhanced resistance against virulent oomycete pathogen *Hyaloperonospora arabidopsidis* (*H.a.*) Noco2. Further genetic analysis showed that mutation in *EDS1* largely suppresses the autoimmunity and elevated SA and NHP biosynthesis in *esd4*, suggesting that loss function of ESD4 leads to EDS1-dependent NLR(s) activation.

## Materials and Methods

### Plant Materials and Growth Conditions

Arabidopsis mutant *line1-12* (*esd4-3*) was obtained from *Agrobacterium*-mediated genetic transformation. *esd4-4* and *fmo1esd4* were generated by CRISPR-cas9 gene editing system (Wang, 2015). *eds1-2* and *fmo1* were previously described (McDowell et al., 2000; Mishina and Zeier, 2006). All the mutants used in this study are in Col-0 ecotype. Col-0 was used as wild-type control and L*er* ecotype was used for generating a mapping population with *line1-12*. As *esd4* mutants are sterile, the heterozygous *esd4* plants were used for producing offspring. The detailed information of mutants used in this study are summarized in Supplementary Table S1. All the primers used for mutant identification are listed in Supplementary Table S2.

For soil-grown plants, seeds were vernalized at 4°C for 2 days, sown onto sterile soil, and transferred to plant growth rooms under 16-h day/8-h night cycle, 22°C, and 65 ± 10% humidity conditions or 16-h day/8-h night cycle, 28°C, and 65 ± 10% humidity conditions. For selecting T1 transgenic plants, sterilized T1 seeds were sown in culture dishes containing 1/2MS medium with hygromycin (40 μg/ml) in growth chamber under 16-h day/8-h night cycle, 22°C and 65 ± 10% humidity conditions.

### Map-Based Cloning and Next-Generation Sequencing

For map-based cloning, *line1-12* homozygote (in Col background) was crossed with L*er* (Landsberg-*erecta*) to generate F1 seeds. Then F1 plants were self-fertilized and the resulting F2 population was used for crude mapping. 64 plants exhibiting dwarfed morphology were picked out from F2 progenies and used for linkage analysis. The markers used for crude mapping were designed using the Monsanto Arabidopsis polymorphism and L*er* sequence collections and listed in Supplementary Table S2 (Jander et al., 2002). After the mutation was narrowed down to a region of about 4.51 Mb between markers T4C9 (6.5 Mb) and F9F13 (11.01 Mb) on Chromosome 4, whole-genome resequencing was performed to identify mutations within this region. 1 g tissue from 52 F2 dwarfed plants from a backcross population between *line1-12* and Col-0 was collected for genomic DNA extraction following a previously described procedure (Huang et al., 2019). The purified DNA was sequenced using Illumina whole-genome resequencing and the indel mutation in *AT4G15880* was confirmed by Sanger sequencing using flanking primers listed in Supplementary Table S2.

### CRISPR/Cas9 Construct Design

The CRISPR/Cas9 constructs for deleting *ESD4* were made following the protocol described previously (Wang, 2015). Briefly, *ESD4* deletion target primers were designed using the CRISPR-PLANT website.^1^ The *ESD4* CRISPR cassette was generated by PCR amplification using pCBC-DT1T2 as template with designed primer pairs. The PCR products were digested with *Bsa*I and cloned into binary vector *pHEE401E*, yielding *ESD4-pHEE401E*. The *ESD4-pHEE401E* construct was transformed into Col-0 or *fmo1* plants by Agrobacterium-mediated floral-dip transformation (Clough and Bent, 1998). Genotyping primers flanking each target site were used to select T1 plants that harbored deletions in *ESD4*. Sanger sequencing was performed to define the *ESD4* deletion. All the primers used are listed in Supplementary Table S2.

### Pathogen Infection Assays

For oomycete pathogen *Hyaloperonospora arabidopsidis* (*H.a.*) Noco2 infection assays, seeds of the indicated genotypes were planted on soil and grown in a growth room under a 16 h day/8 h night cycle, 22°C and 65 ± 10% humidity. Two-week-old seedlings (3 pots for each genotype and 12 plants in each pot) were spray-inoculated with freshly harvested *H.a.* Noco2 conidiospores at a concentration of 1×10^5^ spores per mL sterile water. Infected plants were covered with a plastic lid and kept in a growth chamber under 12-h light/12-h dark cycle, 18°C, and 80% humidity. Seven days later, 1 g plant tissue of wild-type or *esd4* mutant from each pot was collected and suspended in 2 ml sterile water. After vortexing, the conidiospores were counted using a hemocytometer under microscope.

### Trypan Blue Staining and DAB Staining

Trypan Blue staining was performed following a previously described procedure (Zhang et al., 2012). Specifically, true leaves were transferred into 1.5-ml microcentrifuge tubes containing 1 ml lactophenol Trypan Blue solution (10 mg Trypan Blue, 10 g phenol, 10 ml lactic acid, 10 ml glycerol, and 10 ml water) diluted 1:1 in ethanol. After boiling for 2 min, removed staining solution and added 2 ml chloralhydrate solution (2.5 g/ml) on an orbital shaker for 2 h. Samples were further de-stained overnight with new chloralhydrate solution before examination by a light microscope.

DAB staining procedure was performed according to previously described procedures with minor modifications (Daudi and O’Brien, 2012). Briefly, true leaves were transferred into 2.5 ml micro centrifuge tubes containing 2 ml DAB solution (1 mg/ml DAB, pH 3.8). After overnight shaking on an oscillator, DAB solution was removed. 2 ml de-staining solution (70% ethanol) was added for de-colorization. Photos were taken under a light microscope afterwards.

### RNA Extraction and Gene Expression Analysis

Total RNA was extracted from 3-week-old soil-grown plants (50 mg) using Eastep plant RNA extraction kit (Promega, Ls1040). 2 μg RNA was reverse-transcribed to cDNA using Go Script reverse transcriptase (Promega, A5001). 50 ng cDNA was added as template in a 10 μl reaction on an ABI Step one Real-Time system machine. Real-time PCR was performed using a SYBR-Green PCR HS kit (AG, AG11701). qRT-PCR was carried out as described previously (Zhang et al., 2003). *ACTIN1* was used to normalize the expression value. The primers used are listed in Supplementary Table 2.

## Results

### Identification and Characterization of *Line1-12*

**Figure 1:**
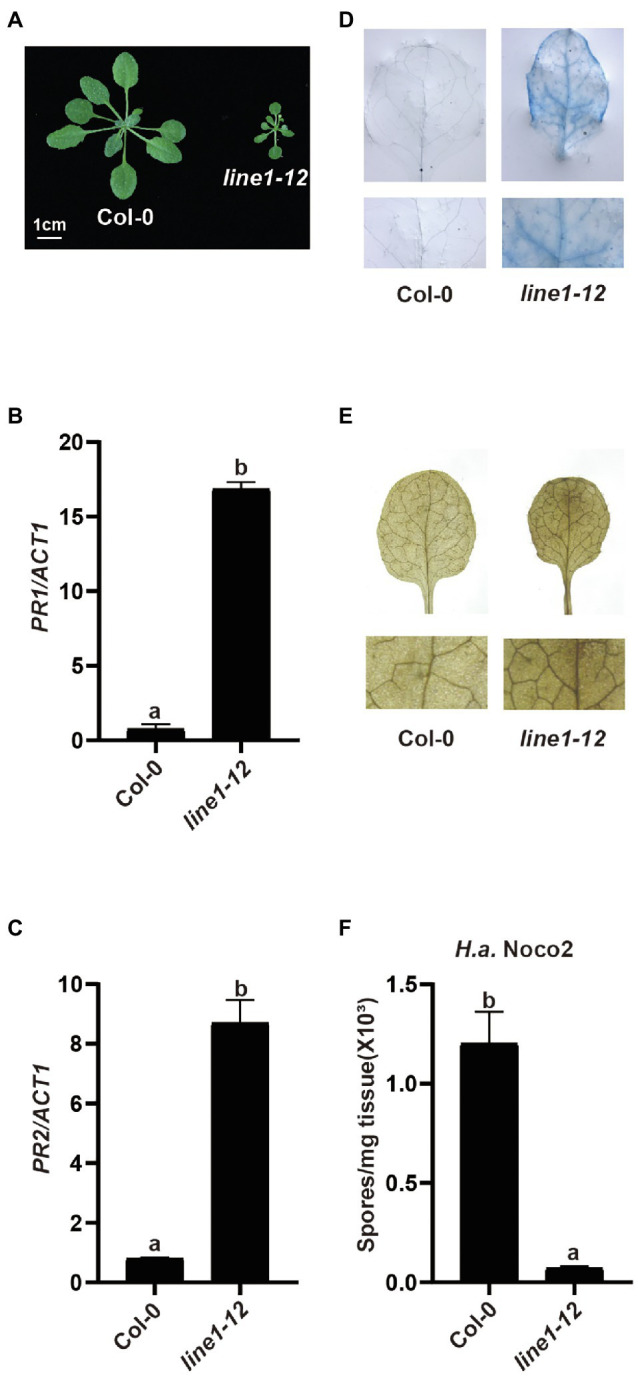
Characterization of *line1-12*. **(A)** Morphology of 3-week-old soil-grown plants of wild-type (Col-0) and *line1-12* under long-day conditions. **(B,C)** Expression of *PR1* **(B)** and *PR2* **(C)** in indicated genotypes. *ACTIN1* serves as internal control. Total RNA was extracted from 3-week-old seedlings grown on soil. Error bars represent means of 3 biological replicates ± SD. Statistical differences among the samples are labeled with different letters (*p* < 0.05, one-way ANOVA; *n* = 3). **(D)** Trypan blue staining of 3-week-old Col-0 and *esd4-3* mutant leaves. **(E)** DAB staining of 3-week-old Col-0 and *esd4-3* mutant leaves. **(F)** Quantification of *H.a.* Noco2 conidia spores on indicated genotypes. 1 × 10^5^ spores/ml inoculum were spray-inoculated on 2-week-old soil-grown seedlings. Numbers of spores were counted at 7 days post-inoculation. Error bars represent means of 3 biological replicates ± SD. Statistical differences among the samples are labeled with different letters (*p* < 0.05, one-way ANOVA; *n* = 3).

With the arrival of better CRISPR-Cas9 gene editing methods in different organisms, knocking out redundant genes has become easier than ever. Designing two guide RNAs (gRNAs) targeting the flanking genes in a tandem array would yield a deletion to knock out these duplicated genes for reverse genetic analysis. Here, we tried to use this system with two gRNAs to knock out a tandem array of 20 genes encoding redundant receptor-like kinases (RLKs) from *At4g23130* to *At4g23320* in the Col-0 ecotype (Wang, 2015). In the T1 generation, among 114 plants tested, we did not detect any deletion products by PCR using primers flanking the two gRNA sites. However, an early flowering dwarfed plant that exhibited abnormal leaf phenotype and sterility was recovered and designated as *line1-12*. Interestingly, *line1-12* resembled mutants with autoimmune defects (Figure 1A). When the defense marker genes *Pathogenesis-Related 1* (*PR1*) and *PR2* were examined by quantitative real-time PCR (qRT-PCR) in *line1-12*, heightened expression was observed (Figures 1B,C). Furthermore, cell death and accumulated H_2_O_2_ were observed in *line1-12* using trypan blue staining and 3,3′-diaminobenzidine staining (Figures 1D,E), respectively. As Col is susceptible to *H.a.* Noco2, this model pathogen on Arabidopsis was used to test for enhanced immunity. When challenged with virulent pathogen *H.a.* Noco2 (Figure 1F), *line1-12* showed less oomycete spores growth compared with wild-type plants. All these data indicate the autoimmunity of *line1-12*. We predicted that the mutation in this mutant should reveal a novel negative regulator of immunity.

### Mutation of *ESD4* Is Responsible for Autoimmunity in *Line1-12*

**Figure 2:**
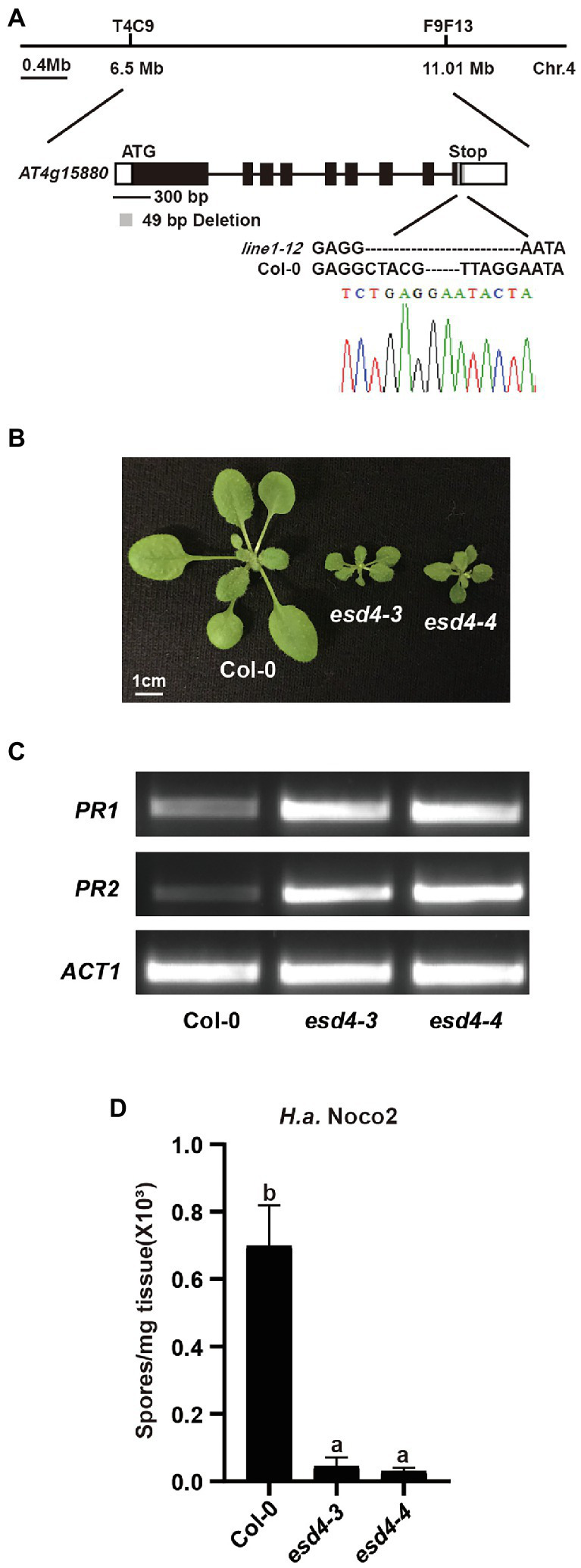
Loss function of ESD4 is responsible for autoimmunity in *line1-12*. **(A)** Mapping-based cloning of *line1-12*. *Line1-12* was mapped to a region between marker T4C9 and F9F13 on chromosome 4. Gene structure of *ESD4* (*AT4G15880*) with *esd4-3* deletion is highlighted in gray. Black boxes and lines indicate exons and introns, respectively. White boxes represent UTRs. Chromatogram of Sanger sequencing confirmed the deletion in *esd4-3*. **(B)** Morphologies of 3-week-old soil-grown plants of the indicated genotypes under long-day conditions. **(C)** Expression of *PR1* and *PR2* in the indicated genotypes. *ACTIN1* serves as internal control. Experiments were repeated three times with similar results. **(D)** Growth of *H.a.* Noco2 on the indicated genotypes. The values indicate averages of replicates ± SD (*n* = 3). Different letters indicate statistical differences among different samples (*p* < 0.05, one-way ANOVA, *n* = 3). Experiments were repeated twice with similar results.

Using map-based cloning, we tested if the mutation responsible for the autoimmunity of *line1-12* is linked to the targeted loci by CRISPR-Cas9. When *line1-12* was crossed with L*er*, 64 dwarfed plants were observed in total 261 F2 population, suggestive of a single recessive mutation. Indeed, the mutation was mapped to chromosome 4 (Figure 2A), where the originally targeted RLK genes are linked to. After failing to find point or small insertion/deletion (Indel) mutations in the target genes through Sanger sequencing, we reasoned that there could be complex rearrangements in the locus. Whole-genome sequencing (WGS) was therefore conducted on the ¼ co-segregants that were dwarfed and flowered earlier in the Col-backcrossed F2 generation. Sequence analysis identified only one mutation, which is an exonic deletion of 49 bp in *At4g15880* in the mapped region (Figure 2A).

*At4g15880* encodes EARLY IN SHORT DAYS 4 (ESD4), which was reported to exhibit dwarfism and early flowering with abnormal leaves and sterility (Reeves et al., 2002; Pineiro et al., 2003; Xu et al., 2007). In addition, *esd4* plants also have increased levels of defense hormone salicylic acid (SA; Villajuana-Bonequi et al., 2014), a well-known defense hormone. Thus, we concluded that the mutant *line1-12* is an allele of *esd4*. As two alleles of *esd4* were reported in the past, we named *line1-12* as *esd4-3*. When the flanking sequences of the deletion in *ESD4* were examined carefully, we did not observe any similarity with the original gRNA sequences, suggesting that the deletion in *ESD4* might be due to CRISPR-Cas9 independent causes, likely through Agrobacterium-mediated mutations.

To further confirm the autoimmune phenotypes of *esd4-3*, we generated another allele of *esd4*, named *esd4-4*, by CRIPSPR-Cas9. *esd4-4* which contains a 2,772 bp deletion in *ESD4* (Supplementary Figure S1A), is also much smaller than wild-type plants and exhibits infertility phenotype under our growth condition (Figure 2B). Semi-qRT-PCR showed that the expression *PR1* and *PR2* were much higher in *esd4-4* compared with that in wild-type (Figure 2C). Similar to *esd4-3*, *esd4-4* showed enhanced resistance to *H.a.* Noco2 (Figure 2D). Taken together, our data further demonstrated that loss function of ESD4 leads to autoimmunity, indicating a negative role of ESD4 in plant immunity.

### FMO1 Contributes to the Autoimmunity of *esd4*

**Figure 3:**
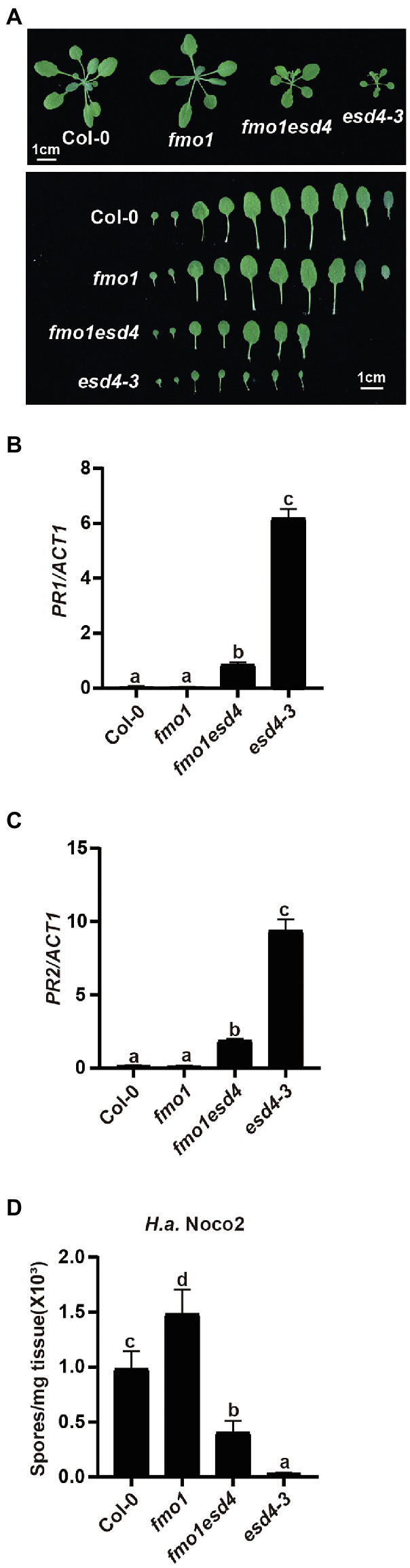
The constitutively activated immunity in *esd4* is partially suppressed by *fmo1*. **(A)** Morphologies of 3-week-old soil-grown plants of the indicated genotypes under long-day conditions. **(B,C)** Expression of *PR1* **(B)** and *PR2* **(C)** in indicated genotypes. *ACTIN1* serves as internal control. **(D)** Growth of *H.a.* Noco2 on the indicated genotypes. The values indicate averages of replicates ± SD (*n* = 3). Different letters indicate statistical differences among different samples (*p* < 0.05, one-way ANOVA, *n* = 3). Experiments were repeated twice with similar results.

Previous study showed *esd4* mutants have increased level of defense hormone SA and SA biosynthesis mutant *ics1* partially reduced the *PR1* expression in *esd4* plants (Villajuana-Bonequi et al., 2014), suggesting that the autoimmunity in *esd4* partly relies on SA signaling and additional factors contribute to the autoimmune phenotypes in *esd4*. As plant signal molecules NHP and SA were reported to cooperatively regulate plant immunity (Sun et al., 2020), we examined whether NHP signaling pathway was involved in the autoimmunity of *esd4*. We generated *esd4 fmo1* double mutant by transforming the CRISPR/Cas9 construct for *ESD4* deletion into *fmo1*, a mutant defective in NHP biosynthesis. A new allele of *esd4*, harboring a deletion of 123 bp, was isolated in the *fmo1* background in T1 generation (Supplementary Figure S1B). As shown in Figure 3A, the *esd4 fmo1* mutant exhibits intermediate size compared to wild-type and *esd4* plant. Upregulated *PR1* and *PR2* levels in *esd4* are also largely suppressed by *fmo1* (Figures 3B,C). In addition, more *H.a.* Noco2 conidiospores grew on the *esd4 fmo1* double mutant compared with *esd4* single mutant (Figure 3D). Taken together, these data showed that *fmo1* partially suppresses the autoimmunity in *esd4*, suggesting that NHP signaling pathway also contributes to autoimmune responses activated in *esd4* mutant.

### Biosynthetic Genes of NHP and SA Are Upregulated in *esd4*

**Figure 4:**
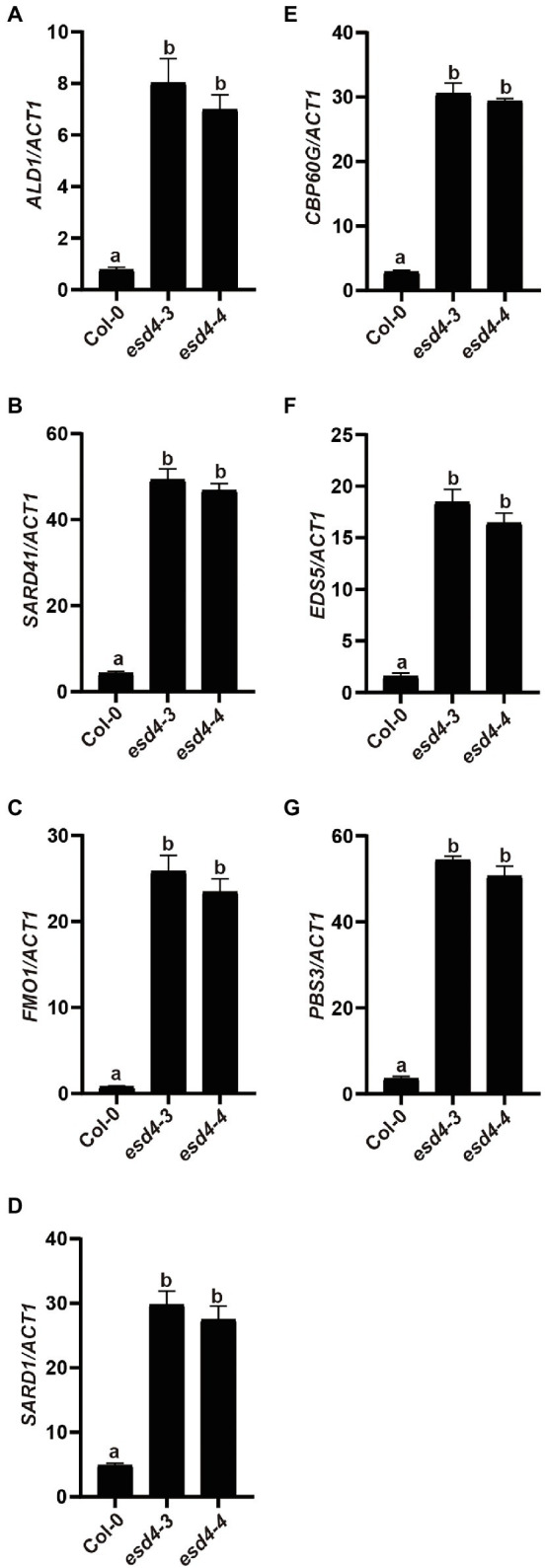
NHP and SA biosynthesis genes are upregulated in *esd4* mutant. qRT-PCR was used to examine the NHP biosynthesis genes **(A-C)**, *SARD1* **(D)**, *CBP60g* **(E)** and SA biosynthesis genes **(F,G)** on three-week-old Col-0, *esd4-3* and *esd4-4* plants. *ACTIN1* serves as internal control. Error bars represent standard deviations. Letters indicate statistical differences (*p* < 0.05, one-way ANOVA, *n* = 3).

To test how NHP signaling pathway contributes to the enhanced disease resistance in *esd4* mutant, expressions of NHP biosynthetic genes were examined. As shown in Figures 4A–C, *ALD1*, *SARD4*, and *FMO1* were all highly upregulated in *esd4*, suggestive of elevated NHP level in *esd4*. NHP biosynthetic genes are coordinately regulated by two master transcriptional regulators SARD1 and CBP60g in *A. thaliana* (Sun et al., 2015). Interestingly, both the expression of *SARD1* and *CBP60g* are greatly induced in *esd4* mutant (Figures 4D,E). SARD1 and CBP60g are also the main regulators of SA biosynthetic genes, including *ICS1*, *EDS5*, and *PBS3* (Zhang et al., 2010; Wang et al., 2011; Sun et al., 2015). Previously, it was shown that *esd4* accumulates much higher SA level compared to wild-type plant and *ICS1* is upregulated in *esd4* mutant under short-day condition (Villajuana-Bonequi et al., 2014). Agreeably, the expressions of other SA biosynthesis genes *EDS5* and *PBS3* are also upregulated in *esd4* mutant (Figures 4F,G). Taken together, these data demonstrate that NHP and SA biosynthesis are upregulated in *esd4*, likely due to the elevated expression of SARD1 and CBP60g.

### EDS1 Is Required for the Autoimmune Phenotypes of *esd4*

**Figure 5:**
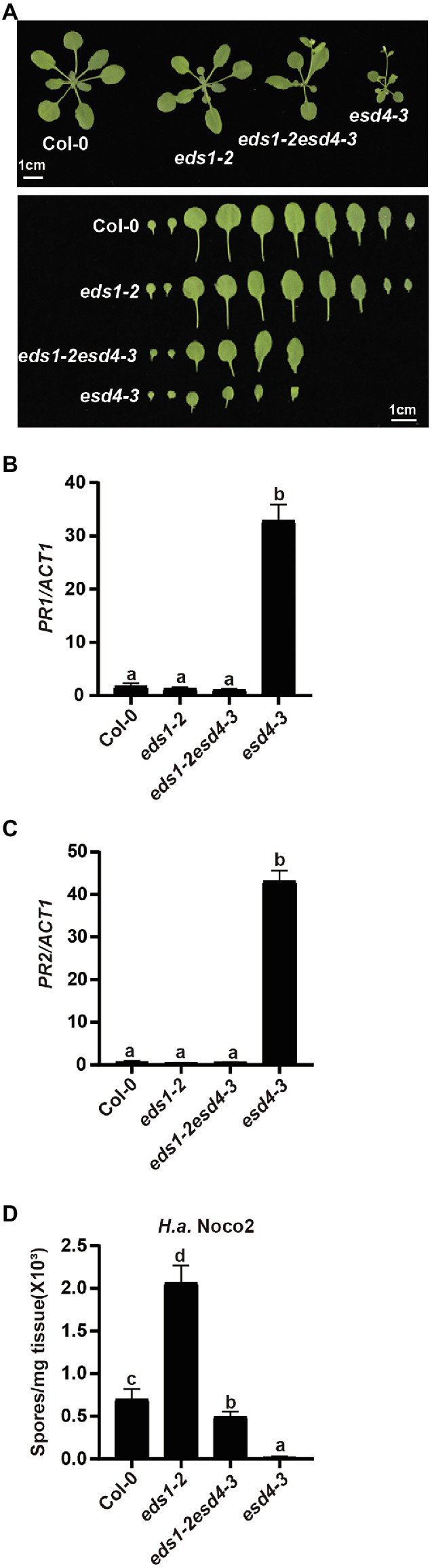
The constitutively activated immunity in *esd4* is suppressed by *eds1*. **(A)** Morphologies of 4-week-old soil-grown plants of the indicated genotypes under long-day conditions. **(B,C)** Expression of *PR1* **(B)** and *PR2* **(C)** in indicated genotypes. *ACTIN1* serves as internal control. **(D)** Growth of *H.a.* Noco2 on the indicated genotypes. The values indicate averages of replicates ± SD (*n* = 3). Different letters indicate statistical differences among different samples (*p* < 0.05, one-way ANOVA, *n* = 3). Experiments were repeated twice with similar results.

EDS1 is a central player downstream of many NLRs and upstream of SA and NHP biosynthesis (Zhang and Li, 2019; Huang et al., 2020). As ESD4 seems to act upstream of SA and NHP biosynthesis, we further examined its relationship with the upstream defense regulator EDS1. *esd4-3* was crossed to loss-of-function mutant of *EDS1*, *eds1-2* (Wagner et al., 2013) to generate *eds1esd4* double mutant. As shown in Figure 5A, the dwarfism of *esd4-3* was largely suppressed by *eds1-2*. Although autoimmunity often results in reduced fertility, *eds1* only mildly restored *esd4* infertility (Supplementary Figure S2). qRT-PCR showed that *eds1-2* also fully blocked the upregulated *PR1* and *PR2* in *esd4-3* (Figures 5B,C). In addition, analysis of resistance to *H.a.* Noco2 indicates that enhanced resistance to oomycete in *esd4-3* was largely compromised by mutation in *EDS1* (Figure 5D). Taken together, these data demonstrate that autoimmunity in *esd4* largely relies on EDS1, indicating that loss function of ESD4 activates NLR-mediated immunity.

### The Elevated SA and NHP Biosynthesis Genes in *esd4* Are Mostly Suppressed by *eds1*

**Figure 6:**
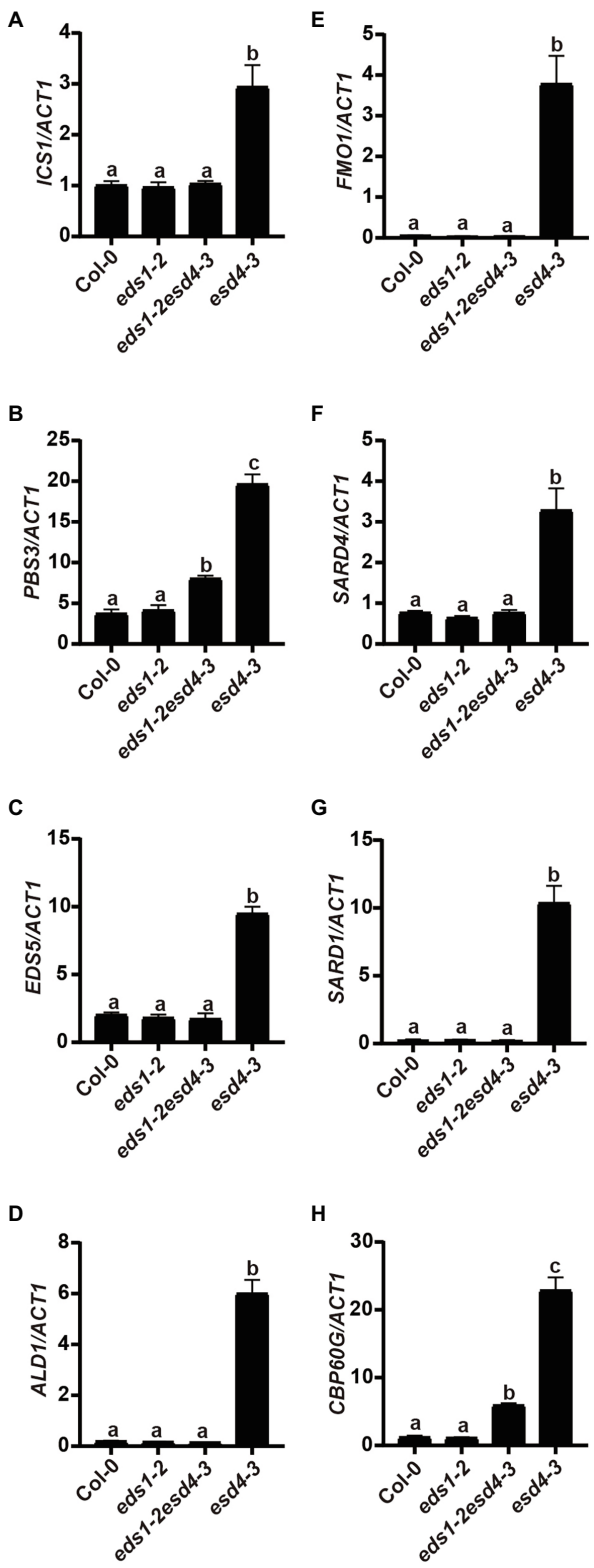
Elevated SA and NHP biosynthetic genes in *esd4* are reduced by *eds1*. qRT-PCR was used to examine the SA biosynthesis genes **(A-C)**, NHP biosynthesis genes **(D-F)**, *SARD1* **(G)** and *CBP60g* **(F)** on three-week-old Col-0, *eds1-2*, *eds1-2esd4-3*, and *esd4-3* mutants. *ACTIN1* serves as internal control. Error bars represent standard deviations. Letters indicate statistical differences (*p* < 0.05, one-way ANOVA, *n* = 3).

Because activation of NLRs can trigger SA and NHP biosynthesis, we then tested whether elevated SA and NHP biosynthesis in *esd4* mutants is due to the activation of certain NLR(s). We measured expressions of SA and NHP biosynthetic-related genes in *eds1esd4* double mutant. As shown in Figure 6, except for *PBS3* and *CBP60g*, all the other highly expressed genes in *esd4* are reduced to wild-type level by *eds1*, further verifying our hypothesis that *esd4* activates NLR-mediated immunity.

Previous studies showed that the autoimmune phenotypes of many autoimmune mutants are temperature-dependent and high temperature often inhibits NLR-mediated signaling. To test whether the autoimmune phenotype observed in *esd4* is temperature-dependent, we grew *esd4* under 28°C conditions. As shown in Supplementary Figure S3, the dwarf morphology and the expression of defense marker genes *PR1* and *PR2* were largely suppressed at 28°C. This is consistent with our finding that activation of NLR(s) leads to autoimmunity in *esd4*.

## Discussion

The plant immune system is elaborate and must be tightly controlled. Insufficient immune activation can lead to susceptibility against pathogens, whereas over-activation under non-pathogenic conditions may result in autoimmunity that impairs plant growth. The typical autoimmune phenotypes are associated with dwarfism, upregulated defense-related genes, spontaneous cell death, elevated SA, and enhanced disease resistance to pathogens. Autoimmunity could be caused by gain-of-function mutations in positive immune receptors/regulators or loss-of-function mutations in negative immune regulators (Van Wersch et al., 2016). SUMO protease ESD4 was initially identified and characterized due to its early flowering phenotype especially under short-day condition (Reeves et al., 2002; Pineiro et al., 2003). In this study, we reported that loss function of ESD4 leads to autoimmunity, suggesting that ESD4 is a negative regulator of plant immunity.

Forward genetic screen under short-day condition designed to search for genes that contribute to early flowering phenotype in *esd4* mutant identified *ICS1*, a major SA biosynthesis gene (Villajuana-Bonequi et al., 2014). Loss of *ICS1* reduces the elevated SA level in *esd4* compared to wild-type, but only suppresses part of upregulated *PR1* in *esd4* mutant under short-day condition. We also analyzed the activated defense response in *sid2 esd4* double mutant under long-day condition and obtained similar results (data not shown), indicative of the partial contribution of SA signaling on the autoimmunity in *esd4*. In parallel of SA, NHP also participates in immune signaling (Bartsch et al., 2006; Liu et al., 2020; Sun et al., 2020). From our analysis of the *fmo1 esd4* double mutant, which is deficient in NHP biosynthesis, we showed that NHP signaling is also required for the autoimmunity in *esd4*.

**Figure 7:**
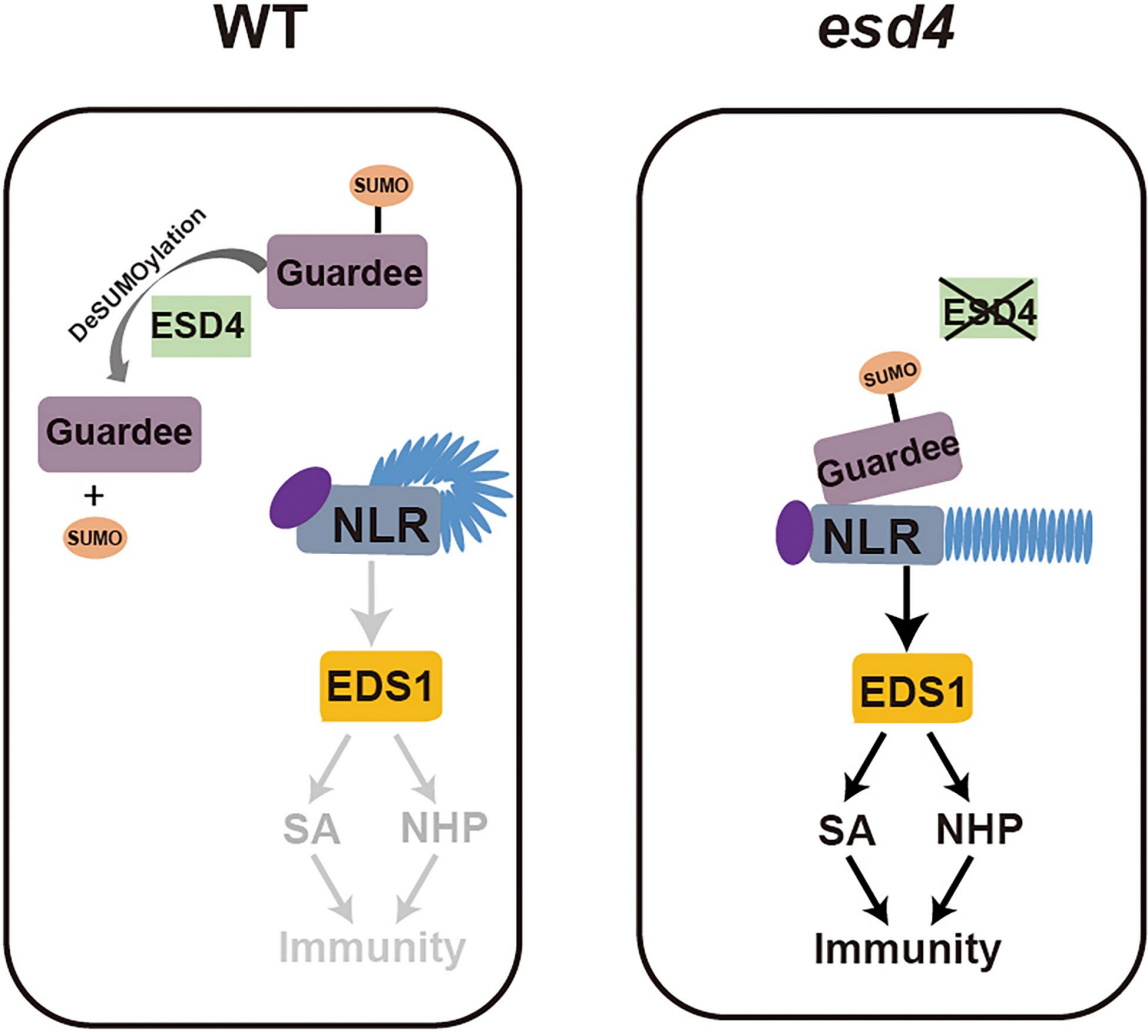
Working model. In wild-type plants, ESD4 deSUMOylates guardee protein to inhibit NLR activation. In *esd4* mutants, the SUMOylating status of guardee is sensed by NLR, leading to the activation of downstream immune signaling including SA and NHP biosynthesis.

From our further epistatic analysis between *esd4* and *eds1*, we revealed that the autoimmunity of *esd4* is largely dependent on EDS1, the immune regulator immediately downstream of many NLR(s), especially for TNLs. This suggests that the autoimmunity of *esd4* is mostly contributed by activation of unknown NLR(s). ESD4, previously reported with SUMO protease activity *in vitro* and *in vivo* (Murtas et al., 2003), is mainly responsible for recycling SUMO from SUMOylated targets, as less free SUMO and more SUMO conjugates than wild-type plants are observed in *esd4* mutant. It is possible that ESD4 directly targets an NLR to remove its SUMO modification for deactivation. However, as there is no evidence on the requirement of SUMOylation in NLR activation, an alternative hypothesis through a guard model may be more plausible. ESD4 was reported to localize to the nucleus and predominantly to the periphery of the nucleus (Murtas et al., 2003). Here the NLR likely guards a host guardee protein *via* its SUMOylation status, which is in turn regulated by ESD4. In wild-type plants, ESD4 removes SUMO from the guardee, preventing its perception by the cognate NLR. In *esd4* mutant plants, the increased SUMOylation of the guardee can be sensed by this NLR, leading to its activation including the upregulation of downstream immune signaling pathways mediated by SA and NHP (Figure 7).

Among the 16 putative SUMO proteases identified in Arabidopsis (Morrell and Sadanandom, 2019), OVERLY TOLERANT TO SALT1 (OST1) and OST2 were also reported to redundantly modulate defense responses *via* SA signaling (Campanaro et al., 2016). Similar to *esd4*, *ots1ots2* double mutants also exhibited autoimmune phenotypes. As highly accumulated SA level and upregulated expression of *ICS1* were observed in *ots1ots2* plants, the constitutively activated immune responses in *ots1ots2* mutants was attributed to the SA signaling. Whether NLR activation leads to the autoimmunity of *ots1ots2*, as in *esd4*, remains to be determined.

The regulations of SA and NHP biosynthesis in plant immunity are complicated (Huang et al., 2020). Even though we proved that the upregulation of SA and NHP biosynthesis observed in *esd4* is largely due to the activation of NLR, we cannot rule out the possibility that ESD4 may directly regulate SA and NHP levels. By sequencing analysis, we found that most of enzymes involved in SA and NHP biosynthesis genes contain either predicted SUMOylated consensus residues (ψKXE/D,ψis a hydrophobic residue; K is a lysine residue; X is any residue; D/E is an aspartic acid or glutamic acid residue) or SUMO interaction motifs (SIMs; Supplementary Figure S4), indicating that they might be targets of ESD4 and their activity or protein level could be directly modulated by ESD4. In addition, some of SA and NHP biosynthesis regulators like SARD1 and CBP60g also have predicted SUMOylated consensus residues and SIMs. Whether they are targets of ESD4 remains unclear. As ESD4 may simultaneously target multiple proteins at different levels, these models can play in orchestra to fine-tune immunity. Identifying the direct targets of ESD4 in the future will shed light on its exact roles in regulating SA and NHP accumulation. In summary, our study provides sufficient evidence demonstrating that loss of SUMO protease ESD4 leads to the autoimmunity-mediated by NLR(s).

## Data Availability Statement

The raw data supporting the conclusions of this article will be made available by the authors, without undue reservation.

## Author Contributions

XL and SX designed the study. XH performed the experiments and prepared the figures and tables. YL and XH wrote the first draft of the manuscript. JH analyzed the whole-genome sequencing data. WF, XL, and SX discussed the results, supervised, and edited the paper. All authors contributed to the article and approved the submitted version.

## Funding

This research is supported by grants from National Natural Science Foundation of China (Grants 30970247 and 31971836) to SX; Natural Sciences and Engineering Research Council of Canada (NSERC) Discovery program received by WF and XL; the Dewar Cooper Memorial Fund from the University of British Columbia to XL. YL is supported by the China Postdoctoral International Exchange Program (Talent-Introduction Program).

## Conflict of Interest

The authors declare that the research was conducted in the absence of any commercial or financial relationships that could be construed as a potential conflict of interest.

## Publisher’s Note

All claims expressed in this article are solely those of the authors and do not necessarily represent those of their affiliated organizations, or those of the publisher, the editors and the reviewers. Any product that may be evaluated in this article, or claim that may be made by its manufacturer, is not guaranteed or endorsed by the publisher.

## Supplementary Material

The Supplementary Material for this article can be found online at: https://www.frontiersin.org/articles/10.3389/fpls.2022.881212/full#supplementary-material

## Footnotes

^1^ http://www.genome.arizona.edu/crispr/CRISPRsearch.html
