# Supplementary material for: Activation of NLR-Mediated Autoimmunity in Arabidopsis Early in Short Days 4 Mutant
Source: Front Plant Sci. 2022 May 25;13:881212. doi: 10.3389/fpls.2022.881212 (PMC9174647; doi:10.3389/fpls.2022.881212)
Supplement: Supplementary file 1 [file Data_Sheet_1.pdf]

Supplemental data

Table 1. Mutants used in this study.

Mutants	Description of mutants	Purpose
line1-12/esd4-3	A 49bp deletion in the last exon of *ESD4*	An *esd4* allele with loss-function mutation of ESD4
esd4-4	A 2772bp deletion in *ESD4* gene	An *esd4* allele with loss-function mutation of ESD4
fmo1	A Salk T-DNA insertion in *FMO1* gene	generating *fmo1esd4* double mutant
fmo1esd4	Knock out *ESD4* in *fmo1* background	Using to reveal ESD4 regulating pathway in plant immunity
eds1-2	A 939bp deletion of *EDS1* gene	Using to generate *eds1esd4* double mutant
eds1-2esd4-3	*eds1-2* crossed with *esd4-3*	Using to reveal ESD4 regulating pathway in plant immunity

Table 2. Primers used in this study.

Primers for vector constructing

primer name	primer sequence (5'-->3')
4g15880-BsF	ATATATGGTCTCGATTGAGATTCATGCTCCTAGTAGGTT
4g15880-F0	TGAGATTCATGCTCCTAGTAGGTTTTAGAGCTAGAAATAGC
4g15880-R0	AACCAGAATCTCTTTAGCTGTTCAATCTCTTAGTCGACTCTAC
4g15880-BsR	ATTATTGGTCTCGAAACCAGAATCTCTTTAGCTGTTC
4g23130-BsF	ATATATGGTCTCGATTGTCCTTCTAACCTTCTTCATGTT
4g23130-F0	TGTCCTTCTAACCTTCTTCATGTTTTAGAGCTAGAAATAGC
4g23320-R0	AACCGCTGCAATCTATCAGCTACAATCTCTTAGTCGACTCTAC
4g23320-BsR	ATTATTGGTCTCGAAACCGCTGCAATCTATCAGCTAC

Primers for map-based cloning

primer name	primer sequence (5'-->3')

T4C9-F	CAAAGGTTTCGTGTCGGAGC
T4C9-R	CGTTGACGGGATACTCGGTG
F9F13-F	CTTGGTTAGTGACCAAGCCTTA
F9F13-R	GATGAATCGTGGATACATTCAGC
FCA2-F	GTTGATGGAACCATCCGAGGATCC
FCA2-R	GGAGCATGGTGCACTCCTCCTAG
T15B16-F	CAAATTTCACTGATTCATCGC
T15B16-R	TGATTGTTCGATTTTGTAGTTG
F8D20-F	TTGATCTGAATAGGTCCCCC
F8D20-R	ACTGTTGCGATAATGCAGTG
F13M23-F	TGATCCCAAGGCCAGCTTAG
F13M23-R	ACTGAGAACTCAGATCG

Primers for genotyping and sanger sequencing

**mutant**	**primer name**	**primer sequence (5'-->3')**
esd4-3	esd4-3-F	cagGTATGACTGTGGAATG
	esd4-3-R	GAGGAAAATATATCGAACCG
esd4-4/5	chesd4-F	CACCGTATTTCCAAGCTTCC
	chesd4-R	CTTCAGCTAGACTAACCAAC
	chesd4-HR	GCTTTCTATAAGCTTCGAGG
eds1-2	eds1-2-g-F	ACAAGCCAAAGTGTCAAGCC
	eds1-2-g-R	CAAGCATCCCTTCTAATGTC
line1	line1-1-F	CGGCTTGCTGAATAATCAC
	line1-1-R	GCTTAGAATACGAAACAGAGG
	line1-2-F	CTGATCGTCCAACGATGTC
	line1-2-R	CTGAGTATCCAAGCATGCAC

Primers for real time qRT-PCR

**primer name**	**primer sequence (5'-->3')**

EDS1-RT-F	GATTATTCAGGTGATCGAGC
EDS1-RT-R	AATCTGCGGTATCGAGTTGC
PR1 F-2	AGGCAACTGCAGACTCATAC
PR1 R-2	TTGTTACACCTCACTTTGGC
PR2-A	GCTTCCTTCTTCAACCACACAGC
PR2-B	CGTTGATGTACCGGAATCTGAC
EDS5-RT-F	GCCAAACAGGACAAGAAAGAAG
EDS5-RT-R	GCCGAAACAATCTGTGAAGC
FMO1-RT-F	GGAGATATTCAGTGGCATGC
FMO1-RT-R	TTTGGTTAGGCCTATCATGG
ALD1-RT-F	TTCCCAAGGCTAGTTTGGAC
ALD1-RT-R	GCCTAAGAGTAGCTGAAGACG
PBS3-RT-F	CTAAGTTCTGGAACTTCTGG
PBS3-RT-R	CATGACTGAAGCAAAGATGG
PAD4-RT-F	CTCTTTCTTCAGTTAAAGATCAAGG
PAD4-RT-R	TAGTGTCCGTACCTCTGATG
ADR1-RT-F	CTTCATACAGGGGAAATGGA
ADR1-RT-R	CAGGAGACATGCCATTGTTG
SARD1-RT-F	CCCTTGCCTCGCCAATTTC
SARD1-RT-R	CAAGTGGATTGTTGTCCACG
CBP60g-RT-F	GAAGAATTGTCCGAGAGGAG
CBP60g-RT-R	GTGAAATCAGCGTTCAGCG
