# Supplementary figures and images for: Activation of NLR-Mediated Autoimmunity in Arabidopsis Early in Short Days 4 Mutant
Source: Front Plant Sci. 2022 May 25;13:881212. doi: 10.3389/fpls.2022.881212 (PMC9174647; doi:10.3389/fpls.2022.881212)

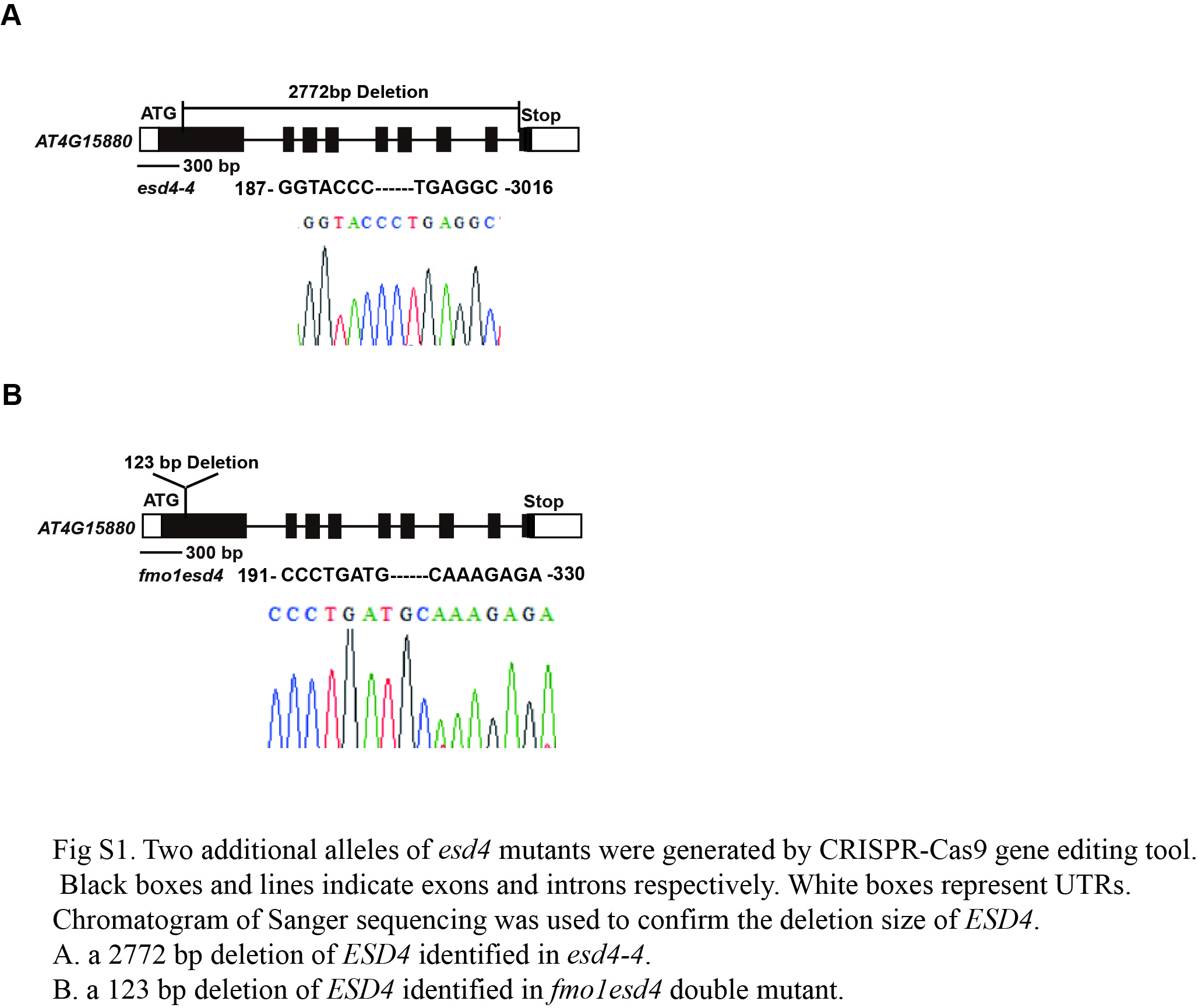

Supplement: Supplementary file 2 [file Image_1.JPEG]

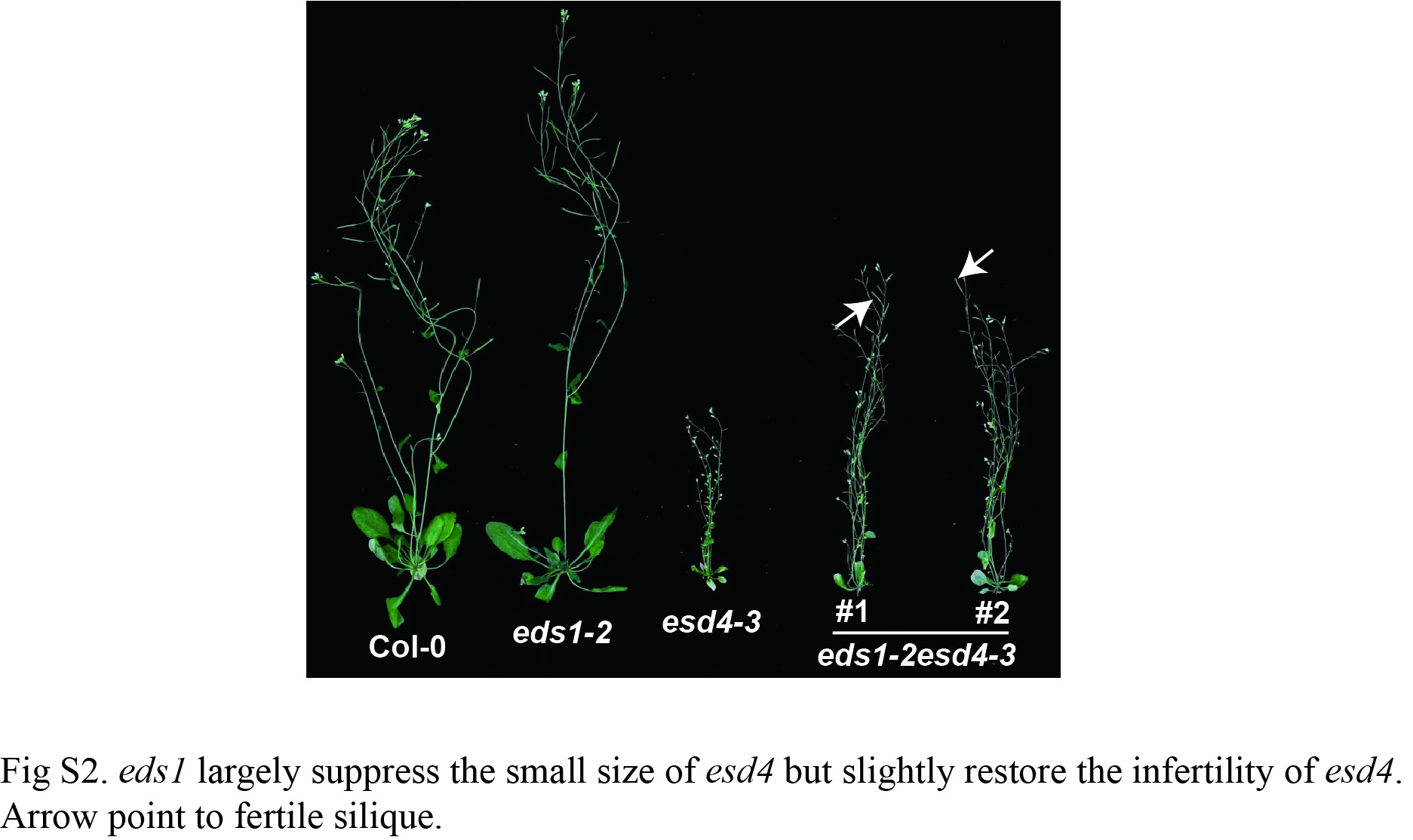

Supplement: Supplementary file 3 [file Image_2.JPEG]

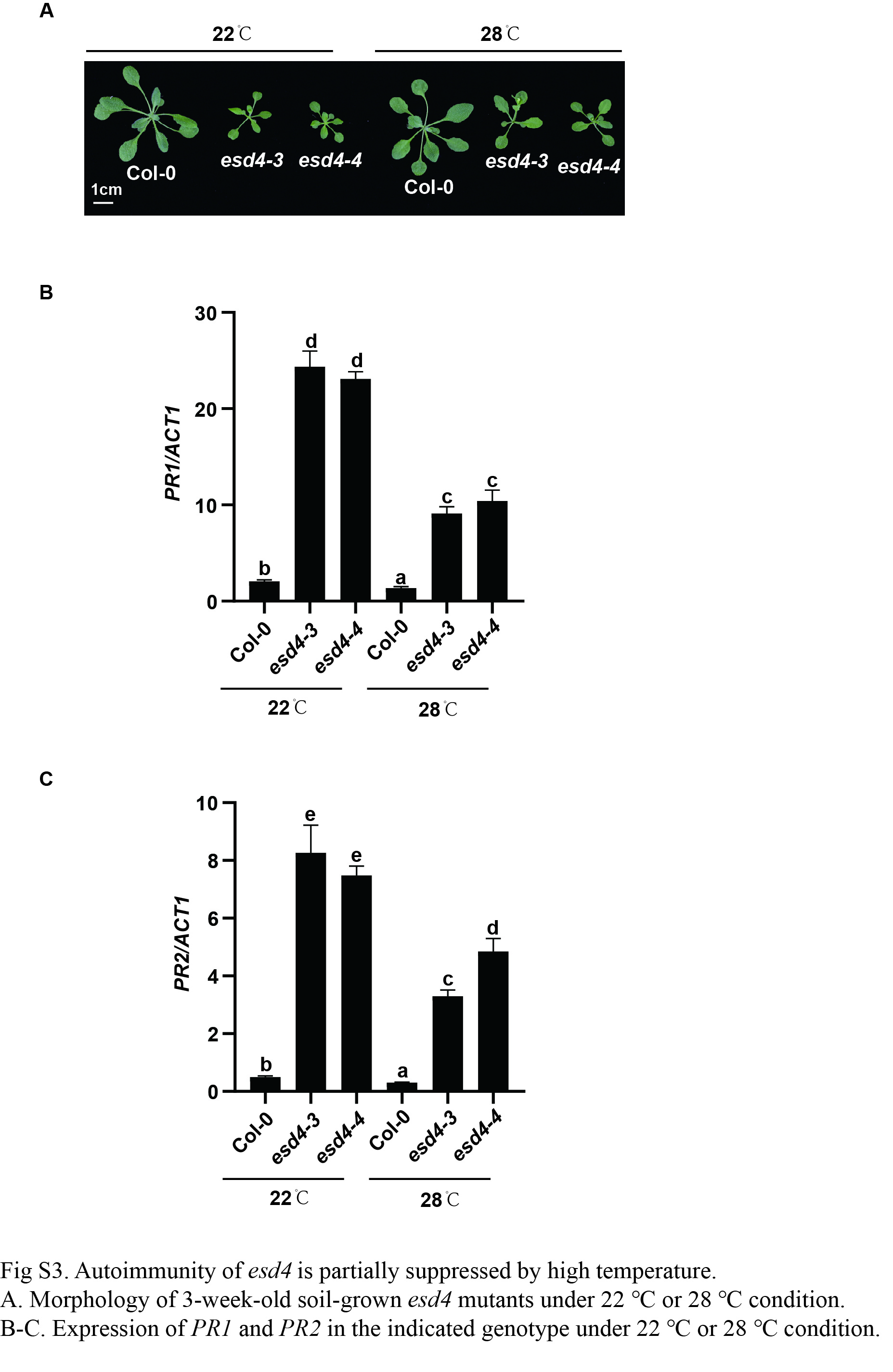

Supplement: Supplementary file 4 [file Image_3.JPEG]

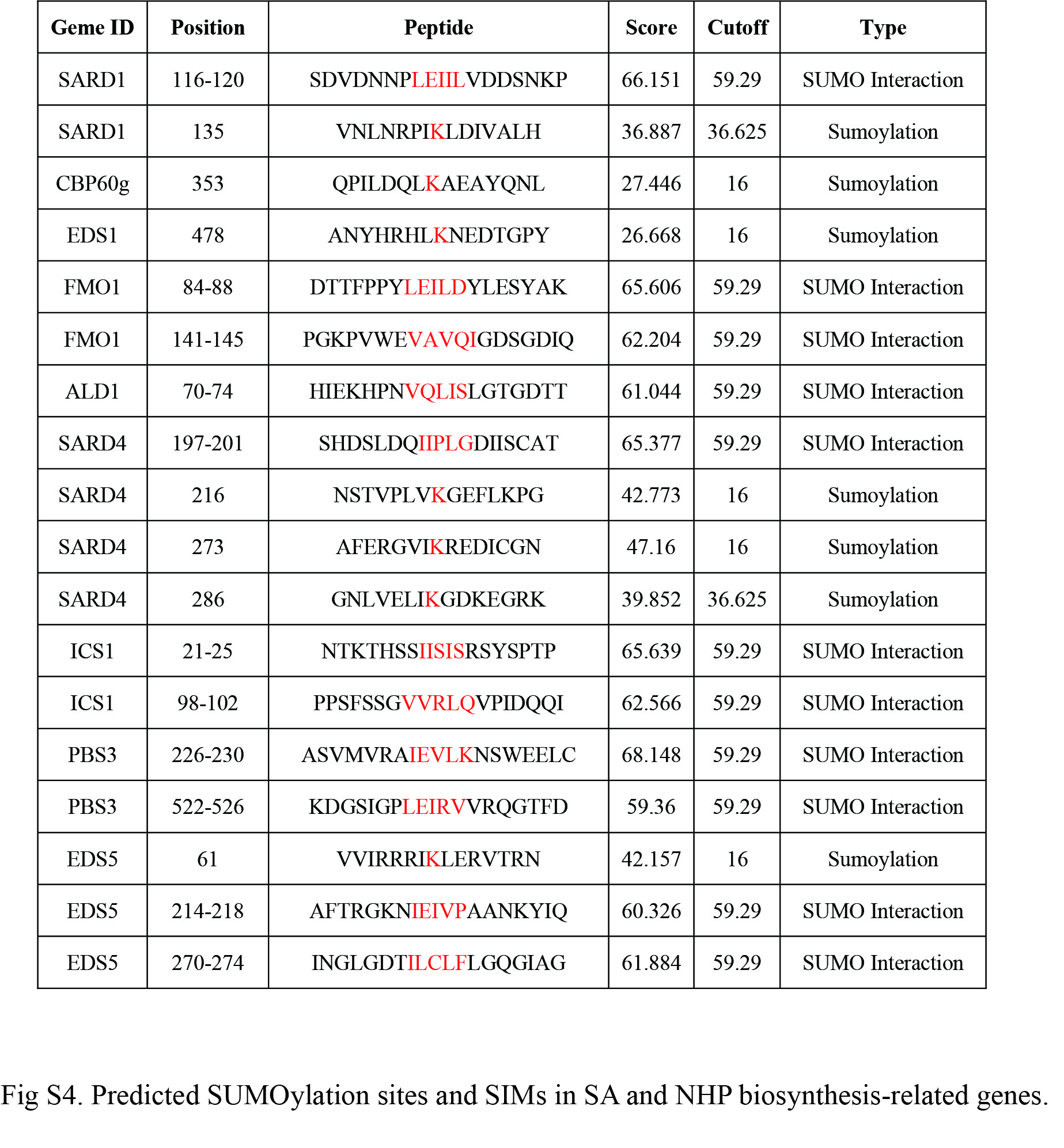

Supplement: Supplementary file 5 [file Image_4.JPEG]
